# Development of a Parsimonious Design for Optimal Classification of Exclusive Breastfeeding

**DOI:** 10.1002/psp4.12428

**Published:** 2019-07-03

**Authors:** Zheng Liu, Aly Diana, Christine Slater, Thomas Preston, Rosalind S. Gibson, Lisa Houghton, Stephen B. Duffull

**Affiliations:** ^1^ School of Pharmacy University of Otago Dunedin New Zealand; ^2^ School of Medicine and Public Health Hunter Medical Research Institute University of Newcastle Rochedale New South Wales Australia; ^3^ Department of Human Nutrition University of Otago Dunedin New Zealand; ^4^ Division of Medical Nutrition Faculty of Medicine Universitas Padjadjaran Bandung Indonesia; ^5^ Independent Consultant Cumbria UK; ^6^ Scottish Universities Environmental Research Centre University of Glasgow Glasgow UK

## Abstract

A deuterium oxide dose‐to‐mother (DTM) technique is used to determine if an infant is exclusive breastfeeding (EBF). However, the DTM method is intensive, requiring seven paired mother–infant samples during a 14‐day study period. The purpose of this study was to develop a field‐friendly protocol. Data from 790 mother–infant pairs from nine countries were analyzed using a Markov chain Monte Carlo method with Stan. The data were split into (i) model building (565 pairs) and (ii) design evaluation (225 pairs). EBF classification was based on a previously published cut‐off for nonmilk water intake. Classification based on the full design was the reference (gold standard classification). The receiver operating characteristics of parsimonious designs were used to determine an optimal parsimonious classification method. The best two postdose windows (days 7–9 and 13–14) yielded optimal categorization with similar performance in the design evaluation data. This postdose two‐sample design provided 95% sensitivity and specificity when compared with the full design.

The World Health Organization recommends exclusive breastfeeding (EBF) of infants for the first 6 months of life for optimal health, growth, and development.^1^ EBF is defined as no food or drink other than breastmilk with the exception of necessary medicines, vitamin and/or mineral supplements, and oral rehydration solutions.^1^ Although the significance of EBF during the beginning of life has been specified as a global health priority, the prevalence of EBF remains low.

In 2012, the 65th World Health Assembly set a global target to increase EBF rates in the first 6 months after birth from 38% to at least 50% by 2025.^2^ As a result, the accurate assessment of EBF rates is critical to evaluating progress. To date, the assessment of EBF at the population level has been based on mother or caregiver reporting, which risks recall and social desirability bias.^3^, ^4^, ^5^ To alleviate this problem, it has become increasingly common to employ more objective methods to quantify EBF using the deuterium oxide (D_2_O) dose‐to‐mother (DTM) technique.^6^, ^7^, ^8^ In this technique, D_2_O is administered orally to the mother, which is then transferred to her child via lactation. The kinetics of D_2_O in the mother and her child are monitored through saliva samples. In our recent work, Liu *et al*.,^9^ a nonlinear hierarchical model was developed describing the kinetics of D_2_O in the mother and her child and identified a criterion for determination of EBF. The criterion was defined by the mass of water intake (denoted *R*_s_) by the infant from sources other than via breastmilk and insensible intake with a value of 86.6 g/day. This value is the lowest mass of water intake that can be determined reliably using the DTM method that can be used as the basis for the classification of mother–infant pairs as EBF or non‐EBF.

Although there are several advantages of the DTM deuterium technique (e.g., relatively noninvasive with known performance characteristics^9^), it is not ideal as the sampling protocol requires intensive sampling for 14 days following a single maternal dose of D_2_O. A less intensive or shorter follow‐up would greatly enhance the utilization of this method for the determination of EBF and non‐EBF characteristics in populations of interest.

The aim of this study was to develop a parsimonious DTM deuterium protocol that classifies mother–infant pairs as EBF or non‐EBF with optimal operating characteristics. It is intended that this protocol could then be taken into future field studies for use in low‐income countries to determine the EBF characteristics in regions of interest and the influence of public health interventions.

## Methods

The methods (and results) are divided into five sections. The data used for this analysis are described in the Data section. The section titled “Model Building and Selection” outlines the model‐building process. “Classification of Mother–Infant Pairs” classifies the mother–infant pairs as EBF or non‐EBF, and “Development of Optimal Allocation Designs” outlines the development and assessment of the streamlined designs. In the section titled “Evaluation of the Selected Streamlined Designs,” the allocation characteristics of the best streamlined design(s) were evaluated via application to a separate data set.

The model for deuterium used in this study was based on our previous work.^9^ Briefly, the best model was described by two‐linked, one‐compartment models (for mother and infant, respectively). The model was built on data that arose from closely controlled data arising from exclusive breastfeeding mothers. The purpose of the model building was to evaluate the estimate of the cut‐off value of water intake from sources other than breast milk (termed *R*_s_) to make the distinction between EBF and non‐EBF in future studies. The identified *R*_s_ cut‐off value was 86.6 g/day, which is used in this study to determine the exclusivity of breastfeeding practices. A summary of the model and the method for calculating *R*_s_ from the parameters are provided in **Supplementary Material** **S1**.

### Data

In this work, the data were available from the following two sources: (i) a calibration study and (ii) a composite field study. The calibration study has been presented in Liu *et al*.^9^ (demographics are provided in **Table** 1 in this article). The composite field study was a combination of a series of studies conducted in eight countries (Sri Lanka, South Africa, Ghana, Thailand, India, Guatemala, Kenya, and Chile). The dosing and sampling methods in all of the studies were based on the protocol described by the International Atomic Energy Agency.^8^ The dose of D_2_O administered to the mothers ranged from 6–60 g as a single dose with the choice dependent on the sensitivity of the measurement techniques used in the particular field study. The measurement of D_2_O was either by isotope‐ratio mass spectrometry or Fourier‐transformed infrared spectrometry. The isotope‐ratio mass spectrometry has greater sensitivity for the measurement of deuterium, and in these studies the D_2_O doses were <30 g, and for the lower sensitivity for the Fourier‐transformed infrared spectrometry studies, the D_2_O dose was ≥30 g.

**Table 1:** Description of the total data and the split data

Contents	Field	Calibration	Model building (two‐thirds field + calibration)	Design evaluation (one‐third field)	All
Subjects, no.	677	113	565	225	790
Dose, g	30 (6–60)	30 (30–30)	30 (6–60)	30 (6–60)	30 (6–60)
Baby age, mo	3.4 (2.9–5.5)	3.3 (2.6–3.7)	3.4 (2.9–5.4)	3.3 (2.9–5.5)	3.4 (2.9–5.5)
Baby WT start, kg	6.4 (5.4–7.3)	5.9 (5.5–6.4)	6.2 (5.5–7.2)	6.3 (5.4–7.4)	6.3 (5.4–7.2)
Baby WT end, kg	6.7 (5.7–7.5)	6.2 (5.8–6.7)	6.5 (5.8–7.4)	6.6 (5.8–7.6)	6.5 (5.7–7.4)
Mother age, y	27 (22–31)	26 (21–30)	26 (21–31)	27 (22–31)	26 (21–31)
Mother WT, kg	58 (51–66)	52 (49–60)	56 (50–64)	58 (53–67)	57 (51–65)
Baby female, %	31	53	36	28	34
Baby male, %	35	47	36	38	36
Baby unknown, %	34	0	27	34	29
IRMS, %	37	0	29	40	32
FTIR, %	62	100	71	60	67
South Africa, %	19	0	15	19	16
Thailand, %	25	0	20	25	22
Kenya, %	28	0	23	28	24
Indonesia, %	1	100	20	0.5	15
Other countries, %^a^	27	0	21	27	23

Row “Dose” is median (range); rows “Baby Age,” “Baby WT Start,” “Baby WT End,” “Mother Age,” and “Mother WT” are median (interquartile range).

FTIR, Fourier‐transformed infrared spectrometry; IRMS, isotope‐ratio mass spectrometry; WT, weight.

Other countries: Sri Lanka, Ghana, India, Guatemala, Chile.

### Model building and selection

All modeling was performed in a fully Bayesian approach using Stan (version 2.12.0; Stan Development Team, New York, NY) with *rstan* (version 2.11.1) interface compiled on C++ (GCC 4.6.3) and run with R (version 3.3.1; R Foundation for Statistical Computing, Vienna, Austria). The structural and statistical models are presented in Liu *et al*.^9^ (Of note, Stan code is available in **Supplementary Material S4**.) Model building here was limited to the further assessment of the residual error models and investigating covariate relationships.

Both additive and combined residual error models were considered, the additive model is presented by Eq. (1) and the combined model by Eq. (2)

(1) $$\text{var}\left(y_{\mathrm{ij}}|{\hat{y}}_{i.j}\right)=\sigma_{1}^{2}$$

(2) $$\text{var}\left(y_{\mathrm{ij}}|{\hat{y}}_{i.j}\right)=f_{\mathrm{ij}}^{2}\sigma_{1}^{2}+\sigma_{2}^{2}$$

where var(.|.) represents the marginal variance of the observation given the model prediction ($\hat{y}$), *y*_*ij*_ is the *j*th observed concentration for the *i*th individual, *f* is the structural model predicted concentration, and σ is the residual standard deviation.

Covariate models were expressed as Eq. (3) for continuous covariates and Eq. (4) for categorical covariates.

(3) $$\ln\left({\overset{¯}{\theta }}_{i}\right)=\ln\left(\theta_{1}\right)+\theta_{2}\ln\frac{{\text{cov}}_{i}}{{\text{cov}}_{\mathrm{median}}}$$

(4) $$\ln\left({\overset{¯}{\theta }}_{i}\right)=\ln\left(\theta_{1}\right)+{\text{cov}}_{i}\ln\left(\theta_{2}\right)$$

where θ_1_ and θ_2_ are the population regression coefficients when a covariate was included in the model, $\ln({\overset{¯}{\theta }}_{i})$ represents the natural log of the expected value for an individual with those characteristics, cov_*i*_ is the covariate value of the *i*th individual, and cov_median_ is the median continuous covariate over the population.

Bayesian analysis settings and the model evaluation criteria in this study are the same as described in our previous work, Liu *et al*.^9^, and are presented in **Supplementary Material** **S2**.

### Classification of mother–infant pairs

Mother–infant pairs were classified as exclusive or nonexclusive on the basis of their individual posterior distributions of *R*_s_ (the parameter that relates to the quantity of water intake rate from sources other than breastmilk). The individual mother–infant pair's posterior distribution of *R*_s_ was determined based on the fit of the best model to the DTM D_2_O data from the model‐building data set (this is also termed the *reference data set*). The criterion defining EBF was determined in our recent work, Liu *et al*.^9^ to be at 86.6 g/day. The determination of exclusivity was according to the following classification:

(5) $$\text{Pr}\;(R_{s}>86.6\;\text{g/day})>0.9\;\text{non-EBF else EBF}$$

Note that the key public health problem depends on accurately determining the nonexclusivity of breastfeeding practice to appropriately focus regional interventions. Hence, we have taken a conservative approach to ensure when classifying a mother–infant pair as non‐EBF, and this is considered at a posterior probability of 90% or greater.

**Figure 1:**
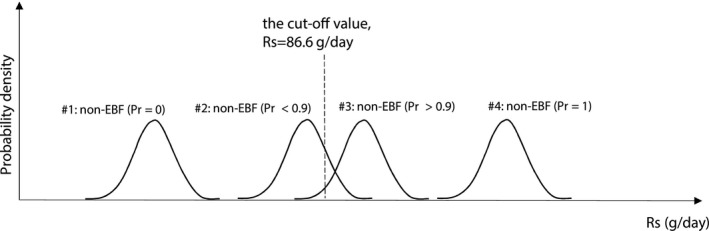
Graphic illustration of exclusive breastfeeding (EBF) or non‐EBF judgment criteria. The probability scale (Pr) is the probability of being non‐EBF (i.e., Pr non‐EBF = Y). Study pair 1 is classified as EBF (i.e., the probability of being non‐EBF is essentially 0), study pair 2 is also classified as being EBF (because the probability of being non‐EBF is < 0.9), study pair 3 is classified as non‐EBF (with Pr > 0.9); and study pair 4 is non‐EBF (with Pr = 1). Rs, water intake rate from sources other than breastmilk.

A graphical presentation of the classification is provided in **Figure** 1.

### Development of optimal allocation designs

We approach the development of parsimonious designs to classify mother–infant pairs as EBF or non‐EBF as an optimal classification problem. The approach was divided into the following two parts: (i) selecting streamlined designs and (ii) determining the operating characteristics of the selected streamlined designs. For the first part, the final model (from the Model Building and Selection section) was fitted to each streamlined design data set, and the posterior distribution of *R*_s_ was computed for each mother–infant pair. In the second part, each mother–infant pair was reclassified as EBF or non‐EBF, and the operating characteristics of the allocation based on the streamlined design were compared to the classification from the reference design. The reference design denotes the full DTM design, which in this work is considered to provide the “gold standard” classification of EBF.

#### Selecting streamlined designs

This work involves the consideration of five data sets. The data sets represented in items 4 and 5 are considered in the evaluation of the streamlined designs.

1. *Model building data set*. This data set consists of the whole calibration data set and two‐thirds of the composite field study data. This was considered the reference (or gold standard) data set. Of note, this data set is unbalanced in terms of the number of mother–infant pairs who provide samples during the duration of the study days.
2. *Imputed full data set*. This is an extension of the model‐building data set with imputed missing data to construct a fully balanced design in terms of the number of samples per individual mother–infant pairs per day. A single imputation method was used where the missing data were imputed with the posterior mean value of the data given the dose of D_2_O, the kinetic model, and the relevant covariates and sample time.
3. *Streamlined data sets*. These represent the combinatorial possibilities of streamlined data sets that could exist in a future field study. Each of these data sets was formed by nonparametric bootstrap from the imputed full data set. The operating characteristics of each of these data sets is considered in a pseudo‐exhaustive search.
4. *Design evaluation data set*. This data set consists of one‐third of the composite field study data. Of note, this data set is unbalanced in terms of the number of mother–infant pairs who provide samples during the duration of the study days.
5. *Imputed full design evaluation data set*. This is an extension of the design evaluation data set with imputed missing data so that it is now fully balanced with regard to the samples per individual per day. A single imputation method was used where the missing data were imputed with the posterior mean value of the data given the dose of D_2_O, the kinetic model, and relevant covariates and sample time.

Streamlined data sets were generated by sampling with replacement from the imputed full data set. Each streamlined data set consisted of the same number of mother–infant pairs as in the model‐building data set but with fewer paired saliva samples from the mother and child. Two types of streamlined designs were considered. The first, streamlined design A, represents a limited sample design where the paired samples could be taken on any day from 1–14. The maximum number of paired saliva samples was set to 3. The second, streamlined design B, represents a limited study duration design (i.e., approximately within a week) where the maximum study duration could be 7, 6, 5, 4, or 3 consecutive study days. In this study, all of the possible examples of the streamlined design (full permutation) were investigated and their operating characteristics were evaluated. Each design was generated once, indicating that the corresponding categorization result was dependent on that local design only.

#### Priors for streamlined designs

It is noted that some streamlined designs will have fewer observations per individual pair than there are structural parameters to estimate (i.e., four parameters per individual model fitting). This results in a situation in which the model would traditionally be considered not identifiable. In a Bayesian sense, this would result in the posterior distribution of the unidentifiable parameters being the same as the prior, i.e., the data would provide no information on particular parameters. In a Markov chain Monte Carlo analysis, this may result in poor convergence properties of the chains. To accommodate this, three different priors could be used in the streamlined analyses.

1. Low‐information prior—using the low‐information prior for the four fitted parameters (i.e., rate constant, describing D_2_O total elimination from the mother compartment (*k*_mm_), D_2_O volume of distribution in mother compartment (*V*_m_), H_2_O clearance rate from mother to infant (CL_mb_) and H_2_O clearance rate from infant (CL_bo_)). This is the same prior as used for the base model analysis. These results are presented in **Supplementary Material** **S3**.
2. Informative prior—using an informative prior for the four fitted parameters. Here, the prior was set to the posterior of the base model analysis. These results are presented in **Supplementary Material S3**.
3. Mixed prior—using an informative prior for *k*_mm_ and *V*_m_ and a low‐information prior for CL_mb_ and CL_bo_. Both CL_mb_ and CL_bo_ are strongly influential in the estimate of *R*_s_ 9, and hence the low‐information prior provides more opportunities for the data to inform their values.

### Streamlined design evaluation

#### Chain stability and convergence

Chain stability and convergence are important criteria for evaluating streamlined designs. The streamlined design is considered to be valid only if the chains appear to superimpose well, indicating good mixing and a stable solution. A detailed discussion for this was presented in Liu *et al*.^9^

#### Operating characteristics of the streamlined designs

It should be noted that these analyses are designed to provide the best estimate of the individual *R*_s_ rather than the population *R*_s_. Hence, any design outcome must consider the correct classification characteristics of the individual.

The characteristics (sensitivity and specificity) of the allocation of mother–infant pairs to EBF or non‐EBF categories for each streamlined design were compared to classification according to the reference design. The characteristics were defined as,

$$\text{Sensitivity}=\frac{\text{True Positives}}{\text{All Positives}}$$

$$\text{Specificity}=\frac{\text{True Negatives}}{\text{All Negatives}}$$

“All Positives” is the total number of non‐EBF pairs, and “All Negatives” is the total number of EBF pairs identified with the reference design. “True Positives” represents the total number of non‐EBF pairs identified with the streamlined design that were also considered non‐EBF by the reference design and the corollary for the “True Negatives.” Streamlined designs A and B can then be compared in terms of their operating characteristics.

## Evaluation of the selected streamlined designs

The streamlined designs A and B were evaluated using the design evaluation data set (consisting of one‐third of the composite data set that was not used in the original model building), with the design evaluation data set providing the reference classification.

The reference classification of mother–infant pairs to the EBF or non‐EBF categories was determined by the following steps. The model (developed from the Model Building and Selection section) was fitted to the design evaluation data set, and the posterior distribution of *R*_s_ was calculated for each mother–infant pair. Each mother–infant pair was then classified as EBF or non‐EBF based on Eq. (5). From the imputed full design evaluation data set, the best streamlined designs A and B were extracted, the model was fitted to these data sets, and their operating characteristics were evaluated and compared to the operating characteristics of those corresponding to streamlined designs A and B.

## Results

### Data

After excluding two pairs as subject outliers (defined as CL_bo_ > 40% of child body weight; see details in Liu *et al*.^9^), the final pooled data set was split into the model‐building data set or the design evaluation data set. The characteristics of the study pairs are summarized and presented in **Table** 1. In total, the model‐building data set contains 565 mother–infant pairs with 6,485 observations. The design evaluation data set contains 225 pairs with 2,481 observations.

### Model building and selection

Additive and combined error models and covariates were tested, and the model performance was evaluated quantitatively and graphically. The criteria for choosing error models, covariates, and model selection are described in **Supplementary Material** **S4**.

The best error model was the combined error model, with country as covariate to accommodate the different assay methods. In addition, mother's weight (MWT) on her volume of distribution (*V*_m_) and infant's weight (BWT) on the CL_bo_ were included in the best final model as presented by Eqs.  (6) and (7).

(6) $$\ln\left(V_{m,i}\right)=N\left(3.54,0.12\right)+N\left(0.46,0.03\right)\ln\frac{{\text{MWT}}_{i}}{70\;\text{kg}}$$

(7) $$\ln\left({\text{CL}}_{\mathrm{bo},i}\right)=N\left(-0.16,0.17\right)+N\left(0.55,0.03\right)\ln\frac{{\text{BWT}}_{i}}{5\;\text{kg}}$$

where *i* is the *i*th individual and 70 kg and 5 kg are the median value of mother's and infant's weights, respectively.

**Figure 2:**
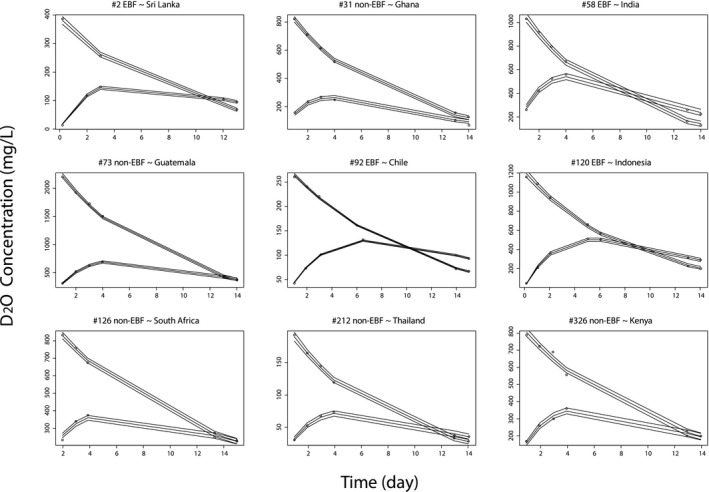
Individual visual predictive check for the best final model evaluation. Open circles are the observations. The solid lines (median, 2.5% and 97.5% quantiles) are the model‐predicted response. D_2_O, deuterium oxide; EBF, exclusive breastfeeding.

The mean and 95% credible interval of each individual's posterior distributions of the parameter values (*k*_mm_ *, V*_m_, CL_mb_, and CL_bo_) and the calculated posterior distribution of *R*_s_ are provided in **Supplementary Material** **S5**. The sampling chains of the best final model were superimposed and well mixed (diagnostics not shown), and Rhat (measure of the ratio of between and within chain variability) values of all the estimated parameters were close to 1.0 (see **Supplementary Material** **S5**), both of which suggest that a stationary solution was found. Individual visual predictive checks (iVPCs) for each mother–infant pair were plotted to assess the final model performance. Each iVPC describes the observations satisfactorily. A total of 31 randomly selected iVPC graphics are displayed, and 9 are presented in **Figure** 2, and the remaining 24 are provided in **Supplementary Material** **S6**. In addition, visual predictive check plots at the population level are also presented in **Supplementary Material** **S6** to illustrate the overall model performance.

### Classification of mother–infant pairs in the model‐building data set (gold standard)

Each mother–infant pair was classified as EBF or non‐EBF based on their posterior distribution of *R*_s_. Classification according to the posterior distribution of the individual *R*_s_ values from this data set was considered the reference (i.e., the gold standard) for computation of the operating characteristics of the streamlined designs A and B. Classification details are provided in **Table** 2.

**Table 2:** Breastfeeding classification by country

Contents	Non‐EBF, n (%)	EBF, n (%)	Total
South Africa	43 (50)	43 (50)	86
Thailand	72 (63)	42 (37)	114
Kenya	31 (24)	96 (76)	127
Sri Lanka	5 (17)	25 (83)	30
India	0 (0)	15 (100)	15
Guatemala	7 (37)	12 (63)	19
Ghana	19 (70)	8 (30)	27
Chile	6 (21)	22 (79)	28
Indonesia	4 (3)	115 (97)	119
All countries	187 (33)	378 (67)	565

EBF, exclusive breastfeeding.

### Development of optimal classification designs

#### Selecting streamlined designs

**Figure 3:**
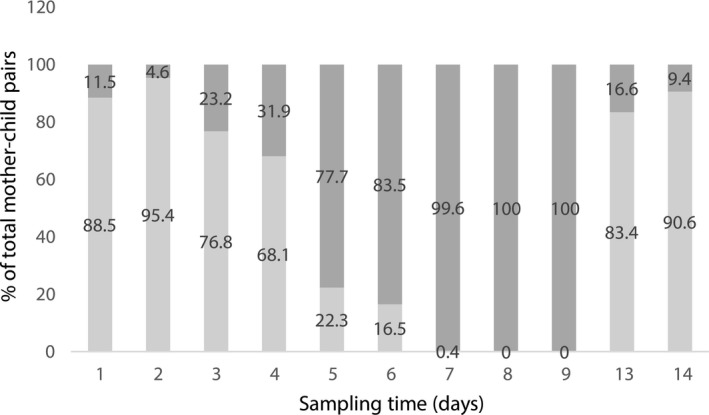
Proportion of the imputed observations vs. the actual measured observations in the imputed full data set. Light gray bars represent the actual (measured) observations; dark gray bars represent the imputed (simulated) observations.

To generate different streamlined designs, a balanced data set is needed. Each individual pair of the 565 mother–infant pairs in the model‐building data set provides an observation for each day. However, the model‐building data set itself is unbalanced, and it cannot be used directly to sample streamlined designs. On the basis of days 1, 2, 3, 4, 5, 6, 7, 8, 9, 13, and 14, the imputed full data set was created. The detailed procedures and rationale of creating the imputed full data set is described in **Supplementary Material** **S7**. **Figure** 3 shows a schematic of the imputed full data set, in which each individual pair of the 565 mother–infant pairs in the model‐building data set provides an observation for each day. Missing values were imputed at their posterior mean. It is assumed that missing values occurred completely at random. The imputed full data set can now be used to sample possible streamlined designs.

#### Applied priors for streamlined designs

Three different priors were considered in this study. Details on the low information and informative prior that were considered are presented in **Supplementary Material** **S3** for comparison. In this study, we concentrated on the mixed prior, which is presented in **Table** 3. The rationale for focusing on the mixed prior is described in the Discussion.

**Table 3:** Mixed prior

Contents	Typical values		BSV	
	Mean	SD	Mean	SD
ln(*V*_m_)	3.45	0.01	0.15	0.01
ln(*k*_mm_)	−2.18	0.01	0.18	0.01
ln(CL_mb_)	0	1,000	0	1,000
ln(CL_bo_)	0	1,000	0	1,000

k_mm_, rate constant, describing D_2_O total elimination from the mother compartment; V_m_, D_2_O volume of distribution in mother compartment; CL_mb_, H_2_O clearance rate from mother to infant; CL_bo_, H_2_O clearance rate from infant. Typical values and between‐subject variability ((BSV) > 0) are assumed as normal distribution, which is denoted here as mean and standard deviation (SD). For ln(*V*_m_), ln(*k*_mm_), and their BSVs, the mean and SD values were set to the posterior of the base model analysis.

#### Streamlined design evaluation

##### Sensitivity and specificity for the streamlined designs

A total of 11 one‐sample designs (i.e., days 1, 2, 3, 4, 5, 6, 7, 8, 9, 13, and 14) were tested with all three priors. All three chains were unstable and have not converged successfully. No single‐sample designs were considered further.

All combinations of permutations (i.e., multiple sample designs) for *s*treamlined design A (where fewer paired samples that could be taken on any day from 1–14) and streamlined design B (where the duration of the study was limited to approximately a week and set to a maximum of 7‐day, 6‐day, 5‐day, 4‐day, or 3‐day studies) with low‐information prior, mixed prior, and informative prior were tested, and the results are presented in **Supplementary Material** **S8**. The priors provided similar design performance. The low‐information prior resulted in chain instability in many runs and was therefore not considered further.

For streamlined design A*,* those promising designs for future field studies with good sensitivity and specificity values (i.e., close to one) are evaluated further for their applicability with the design evaluation data set. For streamlined design B, many of the three‐sample designs performed poorly in terms of sensitivity for the EBF classification. However, the designs with samples that extended to the first full week, e.g., on days 1, 5, or 7, performed satisfactorily. More intensive designs were considered, with many showing good sensitivity and specificity. Of note, only the designs with mixed prior were evaluated.

### Evaluation of the selected streamlined designs

Those potentially favorable streamlined designs with the mixed prior were evaluated against an independent data set that was not used in model building. Each evaluation data set (based on the streamlined designs) was created by sampling with replacement from the imputed full design evaluation data set. The sensitivity and specificity values of these designs from the model‐building data set and the design evaluation data set are listed side by side in **Table** 4. It can be seen that for many designs, the sensitivity and specificity are similar, which supports their potential utility for future field studies.

**Table 4:** Design validation (with the mixed prior)

No.	Sampling days	Sensitivity			Specificity		
		Best^a^	Evaluation^b^	Diff.	Best^a^	Evaluation^b^	Diff.
Streamlined designs A							
1	7 + 13	0.98	0.98	0.00	0.96	0.94	−0.02
2	7 + 14	0.98	0.96	−0.02	0.97	0.94	−0.03
3	8 + 13	0.99	0.98	−0.01	0.96	0.93	−0.03
4	8 + 14	0.99	0.97	−0.02	0.96	0.92	−0.04
5	9 + 13	0.99	0.98	−0.01	0.95	0.93	−0.02
6	9 + 14	0.99	0.98	−0.01	0.96	0.89	−0.07
7	2 + 7+13	0.98	0.99	0.01	0.94	0.88	−0.06
8	2 + 7+14	1.00	0.99	−0.01	0.94	0.89	−0.05
9	2 + 8+13	0.97	1.00	0.03	0.93	0.87	−0.06
10	2 + 8+14	0.99	1.00	0.01	0.93	0.89	−0.04
11	2 + 9+13	0.97	1.00	0.03	0.92	0.85	−0.07
12	2 + 9+14	0.99	1.00	0.01	0.92	0.87	−0.05
13	3 + 7+13	0.99	0.99	0.00	0.94	0.91	−0.03
14	3 + 7+14	1.00	1.00	0.00	0.93	0.86	−0.07
15	3 + 8+13	0.99	1.00	0.01	0.94	0.89	−0.05
16	3 + 8+14	1.00	1.00	0.00	0.93	0.85	−0.08
17	3 + 9+13	0.99	1.00	0.01	0.95	0.89	−0.06
18	3 + 9+14	0.99	1.00	0.01	0.93	0.86	−0.07
Streamlined designs B							
19	5 + 7	0.98	0.99	0.01	0.94	0.89	−0.05
20	5 + 8	0.99	0.99	0.00	0.94	0.89	−0.05
21	5 + 9	0.98	0.99	0.01	0.95	0.89	−0.06
22	1 + 5+7	0.89	0.89	0.00	0.93	0.90	−0.03
23	1 + 5+8	0.91	0.95	0.04	0.94	0.90	−0.04
24	1 + 5+9	0.96	0.99	0.03	0.95	0.92	−0.03
25	2 + 5+7	0.93	1.00	0.07	0.94	0.89	−0.05
26	2 + 5+8	0.95	1.00	0.05	0.95	0.88	−0.07
27	2 + 5+9	0.97	1.00	0.03	0.95	0.87	−0.08
28	3 + 5+7	0.97	0.99	0.02	0.97	0.91	−0.06
29	3 + 5+8	0.99	0.99	0.00	0.97	0.92	−0.05
30	3 + 5+9	0.99	1.00	0.01	0.97	0.92	−0.05

Diff, value of “Evaluation” minus “Best.”

a Best = best streamlined design. These designs were identified and optimized from the imputed full model building data set. They were considered as potentially useful designs for future field studies.

b Evaluation = evaluation of the best streamlined designs. The imputed full model evaluation data set was used to validate the performance of the best streamlined designs.

## Discussion

The currently used sampling protocol of the DTM deuterium technique requires 5–7 paired (mother and infant) saliva samples during a 14‐day study period for the determination of individual breastfeeding practice that is then repeated across a region of interest to gather population characteristics. This requires the healthcare worker to visit the mother (often in remote locations) on several occasions and during the full study period at both significant cost to the study and inconvenience to the family. In this work, a streamlined sampling protocol was developed in which the DTM technique can be used with much less frequent sampling follow‐up to produce a field‐friendly study. Here, it is considered that the streamlined designs that were less intensive in terms of either the number of paired samples or the duration of required study follow‐up. In this work, it is critical to consider the performance of a streamlined design in its ability to correctly assign a mother–infant pair as either EBF or non‐EBF. *R*_s_ is an individual parameter, and the assignment is performed at the individual level. The population characteristics of *R*_s_ are of less importance in this work. Therefore, all designs need to be stable and perform well for the individual.

Among all of the streamlined designs investigated, the two‐sample and three‐sample designs (**Table** 4) performed well for characterizing breastfeeding practice. For streamlined design A (the reduced‐sampling intensity design), it was found that three‐sample designs did not provide significant extra benefits when compared with the two‐sample designs. Of note, the standard deviations of the posterior distribution of *R*_s_ for these streamlined designs were similar between the analyses based on the model‐building data set and design evaluation data set (i.e., at approximately 30–40 g/day). From a practical standpoint, it would be prudent to take an additional convenience sample (yielding a composite three‐sample design that is optimal on two samples) to provide a buffer for execution error in the design.

Studies that addressed reduced study duration were described in streamlined design B, and the intention is to reduce the study duration from 14 days to 9 days as a maximum. The reduced designs nos. 19–21 (two‐sample designs) and nos. 22–30 (three‐sample designs) in **Table** 4 perform equivalently well in terms of sensitivity and specificity. To explore further the shortened protocols, it was considered here that the designs of greater intensity in terms of the number of samples but more parsimonious on study period. The results reveal that some of the designs have good classification performance. However, because of the increased number of samples in total, these designs might have limited use in field studies.

In this work, three priors were considered. However, the use of a mixed‐information prior was primarily investigated and recommended in the future field studies. The intention of this prior is to contribute limited information on the parameters (CL_mb_ and CL_bo_) that have a significant influence on the posterior distribution of *R*_s_ while being informative for those parameters that do not directly influence *R*_s_ (*V*_*m*_ and *k*_mm_). In this setting, the individual's data would “speak” more to the parameters of interest. It is noted that when the information from the data is rich (i.e., the number of samples exceeds the number of parameters), the mixed‐information and low‐information priors performed equivalently. However, in the low‐intensity designs explored here, the mixed‐information prior was required to improve chain stability (it was observed in this study that mixed and informative priors were slightly better than the low‐information prior). Deterministic identifiability (used here to describe the dispersion of the posterior parameter density) together with chain stability are two important issues in this work. The dispersion of the density of *R*_s_ for those mother–infant pairs close to the cut‐off value of 86.6 g/day is a determining factor in their classification as EBF (or otherwise). *R*_s_ is a function of model parameters (which are design dependent) and a number of design‐independent random factors (e.g., absorption of atmospheric water by lungs and skin (*R*_a_), water retaining rate for infant’s growth (*R*_g_), etc., see **Supplementary Material** **S1**). Hence, the mixed prior that provides low information on the model parameters that are informative of *R*_s_ and high information on the uninformative parameters provides a more stable solution. The lower bound on the dispersion of *R*_s_ is given by the dispersion of the design‐independent random variables.

The design framework considered here was based on the optimal assignment of individuals to a dichotomous label given a pseudo‐exhaustive search across potential streamlined designs. The criteria used in this work were based on the evaluation of the receiver operating characteristics of a design when compared with a reference design in terms of sensitivity and specificity. Hence, this work does not directly align with optimal design theory for parameter estimation (for which there are many software available^10^). Although it is possible to optimize the design for the estimation of a subset of the parameters CL_mb_ and CL_bo_, considered the most informative for the calculation of *R*_s_, there is no guarantee that these designs would perform equivalently for the calculation of *R*_s_ and assignment to the EBF or non‐EBF criteria. The design space was explored initially by assessing the sensitivity of the estimate of CL_mb_ and CL_bo_ to the sampling design (**Supplementary Material** **S7** **Figures S10 and S11**) and found that the maximum information density of CL_mb_ and CL_bo_ occurred at sampling times day 4.5 and day 8. It was noted that the best two‐sample designs required the first sample from days 7–9 and the second sample from days 13 or 14 as shown in nos. 1–6 of **Table** 4. It appears that the high sensitivity of the day 8 sample may be reflected in the two‐sample designs; however, there is no clear alignment between the sensitivity of the design and that found to be optimal for classification.

In this study, sample imputation was needed to create the imputed full data set and consequently reduced to the streamlined data sets of interest. In this setting, a streamlined design was sampled nonparametrically from the imputed full data set for the assessment of its operating characteristics, and this sampling was conducted separately for each mother–infant pair. The influence of potential artefacts in the sampled designs across the population was not assessed and in practicality could not be assessed given the pseudo‐exhaustive search conducted in this work. However, because a Bayesian framework was used in which the full posterior distributions of the parameters were estimated for each individual then the influence of a possibly erroneous data combination would have been reflected in the uncertainty of the parameters that would affect the classification to non‐EBF on a case‐by‐case basis. Given the performance criteria, sensitivity and specificity were based on the reference design (without imputation), and it is believed that the influence of any erroneous data would have been negligible.

The ideal approach for this study would be to have data that arise from controlled EBF and controlled non‐EBF mother–infant pairs. This would then act as the gold standard from which full and reduced designs can be evaluated to evaluate the correct categorization of EBF or non‐EBF. This approach is, however, not possible because controlled non‐EBF data would require infants to be weaned for the purpose of the study, placing the infant at considerable risk, which would not be clinically or ethically appropriate. Our approach is to use an accepted model for deuterium DTM analysis^6^, ^7^, ^8^ as the basis for decisions on the operating characteristics of EBF designs and applying these to the uncontrolled model‐building and design evaluation data sets.

## Conclusions

The parsimonious designs identified in this work are intended to be applied in field studies and prospectively tested. When compared with the currently used 14‐day studies that require 5–7 sample designs, the streamlined designs identified here have clear advantages in terms of convenience for the mothers, their families, and the research staff. It is envisaged that the benefits of this work will improve the knowledge of the non‐EBF characteristics of regional populations that could then lead to targeted intervention programs.

## Conflict of Interest

The authors declared no competing interests for this work. As an Associate Editor for *CPT: Pharmacometrics & Systems Pharmacology*, Stephen Duffull was not involved in the review or decision process for this article.

## Author Contributions

Z.L., L.H., and S.B.D. wrote the manuscript. All authors designed the research. Z.L. and S.B.D. analyzed the data. A.D., C.S., T.P., R.S.G., and L.H. contributed new reagents/analytical tools.

**Study Highlights**

**WHAT IS THE CURRENT KNOWLEDGE ON THE TOPIC?**

☑ The advantages of exclusive breastfeeding are well known. The deuterium oxide dose‐to‐mother (DTM) technique is an objective method to quantify exclusive breastfeeding (EBF) practice and to classify the mother–infant pair as EBF or non‐EBF. However, the DTM protocol is intensive and not field friendly, requiring seven postdose samples during a 14‐day study period.

**WHAT QUESTION DID THIS STUDY ADDRESS?**

☑ This study builds on previous work where a cut‐off value for non–milk‐based water intake was determined. In this work, a series of field‐friendly streamlined designs were developed for optimal classification of mother–infant pairs as EBF or non‐EBF.

**WHAT DOES THIS STUDY ADD TO OUR KNOWLEDGE?**

☑ As an example of an optimal classification experiment, these designs identified and optimally classified mother–child pairs into EBF or non‐EBF categories, which has important population‐level public health implications.

**HOW MIGHT THIS CHANGE DRUG DISCOVERY, DEVELOPMENT, AND/OR THERAPEUTICS?**

☑ As an important field tool, the parsimonious designs will minimize the burden of field studies and allow for the improved monitoring and evaluation of breastfeeding rates and the evaluation of public health support strategies.

## Supporting information


**Supplementary Material S1.**

**Supplementary Material S2.**

**Supplementary Material S3.**

**Supplementary Material S4.**

**Supplementary Material S5.**

**Supplementary Material S6.**

**Supplementary Material S7.**

**Supplementary Material S8.**
