# Supplementary material for: Development of a Parsimonious Design for Optimal Classification of Exclusive Breastfeeding
Source: CPT Pharmacometrics Syst Pharmacol. 2019 Jul 3;8(8):596–605. doi: 10.1002/psp4.12428 (PMC6709417; doi:10.1002/psp4.12428)
Supplement: Supplementary file 1 — Supplementary Material S1. [file PSP4-8-596-s001.pdf]

Supplement 1.

Of note, the content in this supplement serves as a brief summary and the corresponding details were presented in our previous publication [9].

Structure model

The structure model used in this study is a two linked 1-compartment disposition models that represent mother and infant as in Figure S1.

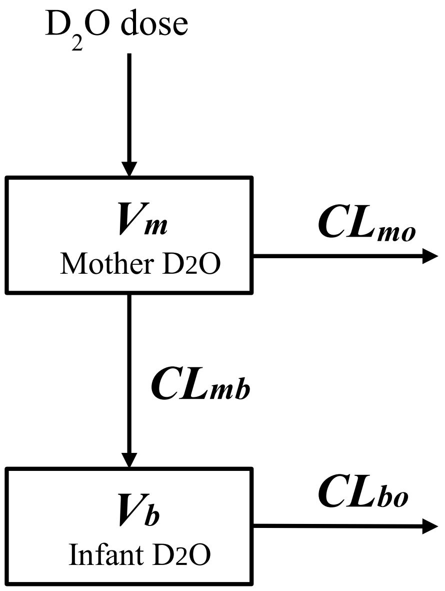

Figure S1. D_2O disposition model for mother and infant. The term V denotes the D_2O volume of distribution with subscript m and b for mother and baby (also termed infant); CL_{mb} is the water clearance from mother to infant; CL_{bo} is the water clearance from infant to out; the term CL_{mo} represents the water clearance from mother to out.

The system expressed as rate constants (for simplicity) is given:

$$dA_m/dt = -(k_{mm} - k_{mb})A_m - k_{mb}A_m = -k_{mm}A_m \qquad (\text{at } t = 0,\ A_m = dose) \qquad (1)$$

$$dA_b/dt = k_{mb}A_m - k_{bo}A_b \qquad (\text{at } t = 0,\ A_b = 0) \qquad (2)$$

The analytical solutions of equation (1) and (2) are:

$$A_{m(t)} = A_{m(0)}e^{-k_{mm}t} \qquad (3)$$

$$A_{b(t)} = A_{m(0)}\left(\frac{k_{mb}}{k_{mm}-k_{bo}}\right)\ (e^{-k_{bo}t} - e^{-k_{mm}t}) \qquad (4)$$

Where,

$$k_{bo} = k_{bo(H_2O)} = \frac{CL_{bo}}{V_b} \qquad (5)$$

$$k_{mb} = k_{mb(H_2O)} = \frac{CL_{mb}}{V_m} \qquad (6)$$

In this notation, $A_{m(t)}$ is the mass of D_2O in mother compartment at time t, (units: kg); $A_{m(0)}$ is equal to dose, (units: kg); $A_{b(t)}$ is the mass of D_2O in infant compartment at time t, (units: kg); k_{mm} is the rate constant, describing D_2O total elimination from the mother compartment, (units: 1/day); k_{mb} is the rate constant describing D_2O flow from the mother to the infant via lactation route, (units: 1/day); $k_{mb(H_2O)}$ is the rate constant describing H_2O flow from the mother to the infant via lactation route, (units: 1/day); k_{bo} is the rate constant describing D_2O flow out of the infant compartment, (units: 1/day); $k_{bo(H_2O)}$ is the rate constant describing H_2O flow out of the infant compartment, (units: 1/day). CL_{bo} is the H_2O clearance rate from infant, (units: L/day); CL_{mb} is the H_2O clearance rate from mother to infant, (units: L/day); V_b is the D_2O volume of distribution in infant compartment, (units: L); and V_m is the D_2O volume of distribution in mother compartment, (units: L).

The final model consists of four parameters k_{mm}, V_m, CL_{mb} and CL_{bo}. Of these, the parameters CL_{mb} and CL_{bo} are of primary importance to determine the non-breastmilk water intake.

Statistical models

A standard three-stage hierarchical model was used. Stage 1) the model for the data; Stage 2) the model for heterogeneity between individuals; Stage 3) the model for the priors. An additional part is also presented here about the statistical models to calculate R_s.

Stage 1: model for the data

$$y_{ij} \sim N(f(\boldsymbol{\theta_i}, x_{ij}),\ \sigma^2) \quad (7)$$

where y_{ij} denotes the jth observation for the ith subject, $f(\boldsymbol{\theta_i}, x_{ij})$ is the expected value of the data from the model prediction, $\boldsymbol{\theta_i}$ is a vector (dimension $p \times 1$, where p is the number of parameters) of individual parameter values for the ith individual, x_{ij} is a sampling time (and other design variables such as dose), N represents a normal distribution with (in this case) zero mean and standard deviation σ.

Stage 2: model for heterogeneity between individuals

The distribution of an individuals' PK parameter vectors $\boldsymbol{\theta_i}$ are shown,

$$\boldsymbol{ln}(\boldsymbol{\theta_i}) \sim N_p(\ln(\boldsymbol{\mu}), \boldsymbol{\Omega}), \text{ and} \quad (8)$$

$$\boldsymbol{\Omega} \sim Q_p(\boldsymbol{\rho}, \mathbf{V}) \quad (9)$$

where $\boldsymbol{\mu}$ is a vector of mean population pharmacokinetic parameters and $\boldsymbol{\Omega}$ is the variance–covariance matrix of between subject random variability. N_p represents a $p-$dimensional multivariate normal distribution. Q_p is the quadratic form using the column vector $\mathbf{V}$ as a diagonal matrix, $\boldsymbol{\rho}$ is the LKJ correlation matrix, generating random correlation matrices based on vines and extended onion method [11]. Q_p is equivalent with the calculation result of $\mathbf{V\rho V}$

(where V is diagonal), which provides the variance-covariance matrix for the fitted parameters. A detailed description about $\boldsymbol{\rho}$ and $\mathbf{V}$ can be found in [12].

Stage 3: Model for the priors

Priors for the analysis include: 1) priors for the parameters and, 2) priors for the known variables.

The prior of the residual variance is:

$$\sigma \sim N(0, 1000) \text{ with } \sigma > 0 \qquad (10)$$

Here σ is sampled from a truncated normal distribution.

The prior for the vector of mean parameters, in this study, $\boldsymbol{\mu}$, i.e. CL_{mb}, CL_{bo}, k_{mm},and V_m, is given by a low information prior was assumed for all:

$$\ln(\boldsymbol{\mu}) \sim N(0, 1000) \qquad (11)$$

The priors of the variance–covariance matrix $\boldsymbol{\Omega} \sim Q_p(\boldsymbol{\rho}, \mathbf{V})$ is:

$$\boldsymbol{\rho} \sim \text{lkj\_corr}(1) \qquad (12)$$

$$\boldsymbol{\lambda}_{\mathbf{i}} \sim N(0, 1000) \text{ with } \boldsymbol{\lambda}_{\mathbf{i}} > 0, \text{ and } \mathbf{V} = \boldsymbol{\lambda I_p} \qquad (13)$$

Here I_p represents a $p \times p$ identity matrix, the parameter "1" in the lkj_corr function is the shape parameter. In this case "1" represents a bounded uniform distribution on the space of correlations, and $\mathbf{V}$ is from a truncated normal distribution.

Calculation of R_s

$$R_s = R_{c(bo)} + R_g - CL_{mb} - R_m - R_a \qquad (14)$$

The models used to calculate $R_{c(bo)}$, R_g, R_m and R_a are presented as below. NB, the value of CL_{mb} is part of the structural model for which the posterior distribution is estimated.

- $R_{c(bo)}$: Total infant's water output rate after isotopic fractionation correction

$$R_{c(bo)}(L/day) = CL_{bo}/0.9906 \qquad (15)$$

NB, the value of CL_{bo} is part of the structural model for which the posterior distribution is estimated.

- R_g: Water retaining rate for infant's growth

$$R_g(L/day) = (TBW_{ls} - TBW_{fs})/(day_{ls} - day_{fs}) \quad (16)$$

TBW_{ls} and TBW_{fs} represent the infant's total body water at the last sampling day (i.e. subscript *ls*) and at the first sampling day (i.e. subscript *fs*), respectively and $day_{ls} - day_{fs}$ describes the experimental sampling period, usually 14 days in this study.

TBW is calculated with equation (17), according to the infant's weight, WT.

$$lnTBW\ (L) = a\ +\ (b \times lnWT) \quad (17)$$

Where,

$a \sim N(-0.427, 0.012)$
$b \sim N(0.963, 0.005)$

- CL_{mb} – a structural parameter in the pharmacokinetic model.

- R_m: Intake rate of water metabolised from protein, fat, and carbohydrate in breastmilk

$$M(kg/day) = CL_{mb}/[0.889\ (\text{SE}, 0.0013)] \quad (18)$$

$M(kg/day)$: the total mass of breastmilk intake per day

$$R_m(L/day) = \ [0.07733\ (\text{SE}, 0.00117)]\ \times M \quad (19)$$

- R_a: Absorption of atmospheric water by lungs and skin

$$R_a(L/day) = \ [0.063\ (\text{SE}, 0.017)] \times (R_{c(bo)} + R_g) \quad (20)$$
