# Supplementary material for: Development of a Parsimonious Design for Optimal Classification of Exclusive Breastfeeding
Source: CPT Pharmacometrics Syst Pharmacol. 2019 Jul 3;8(8):596–605. doi: 10.1002/psp4.12428 (PMC6709417; doi:10.1002/psp4.12428)
Supplement: Supplementary file 2 — Supplementary Material S2. [file PSP4-8-596-s002.pdf]

Supplement 2.

Bayesian analysis settings

Models were parameterized in terms of the natural log of the parameters values [13] (e.g. *ln* (CL), *ln*(*V*)). In the present study, three MCMC chains were run simultaneously. Each MCMC chain was run for 10000 samples (excluding the 1000 samples that were discarded during the burn-in phase). The three MCMC chains were pooled to represent the posterior distributions of the parameter values of interest.

Model Evaluation

The initial estimates of all the chains were selected by Stan randomly. Convergence of the MCMC chains were assessed using the potential scale reduction factor, Rhat (a measure of the ratio of between and within chain variability). All MCMC chains were assumed to have reached the stationary distribution if Rhat values were close to 1.0 for all parameters [14]. Furthermore, the trace history of MCMC samples for all chains were examined visually for all parameters, in which a 'fuzzy caterpillar' [15] suggests that MCMC chains had reached a stationary distribution. In addition, the number of effective samples in a chain "n_eff" [16] was used to investigate the sampling efficiency (i.e. the number of independent samples) during the analysis for computation of summary measures. In addition, visual predictive checks based upon individual mother-infant pair observations were available for evaluation.
