# Supplementary material for: Development of a Parsimonious Design for Optimal Classification of Exclusive Breastfeeding
Source: CPT Pharmacometrics Syst Pharmacol. 2019 Jul 3;8(8):596–605. doi: 10.1002/psp4.12428 (PMC6709417; doi:10.1002/psp4.12428)
Supplement: Supplementary file 3 — Supplementary Material S3. [file PSP4-8-596-s003.pdf]

Supplement 3.

Table S1. Low information prior

	typical values		BSV	
	mean	SD	mean	SD
ln(V_m)	0	1000	0	1000
ln(k_{mm})	0	1000	0	1000
ln(CL_{mb})	0	1000	0	1000
ln(CL_{bo})	0	1000	0	1000

*typical values and BSV (Between Subject Variability; BSV>0) are assumed as normal distribution, which is denoted here as mean and standard deviation (SD).

Table S2. Informative prior

	typical values		BSV	
	mean	SD	mean	SD
ln(V_m)	3.45	0.01	0.15	0.01
ln(k_{mm})	-2.17	0.01	0.18	0.01
ln(CL_{mb})	-0.36	0.02	0.31	0.01
ln(CL_{bo})	-0.05	0.02	0.23	0.01

*typical values and BSV (Between Subject Variability; BSV>0) are assumed as normal distribution, which is denoted here as mean and standard deviation (SD). For all the parameters, the mean and SD values were set to the posterior of the base model analysis.
