# Supplementary material for: Development of a Parsimonious Design for Optimal Classification of Exclusive Breastfeeding
Source: CPT Pharmacometrics Syst Pharmacol. 2019 Jul 3;8(8):596–605. doi: 10.1002/psp4.12428 (PMC6709417; doi:10.1002/psp4.12428)
Supplement: Supplementary file 4 — Supplementary Material S4. [file PSP4-8-596-s004.pdf]

Supplement 4.

Different error models (additive and combined error models) and covariates (baby's weight and age, mother's weight and age, country and assay methods) were tested. The criteria used were the Watanabe-Akaike information criterion (WAIC) and Leave One Out (LOO) Cross Validation. Detailed definitions of WAIC and LOO were presented in Liu et al. [9]. The values of WAIC and LOO of different models in this study are presented in Table S3.

The combined error model was statistically preferred to the additive error model according to the WAIC and LOO values, as well as the graphic visual check. Incorporation of country as covariate to the combined error model was also statistically preferred. Including country as a covariate on the residual error model accounted for both regional differences as well as differences in assay procedures (e.g. IRMS or FTIR), as in each country only one assay was used. It is noted that addition of mother's weight on V_m; baby's weight on CL_{bo} agree with the marginal correlations (Figures S2 and S3), but were not preferred statistically according to both WAIC and LOO, nevertheless these covariates were retained due to their biological plausibility.

Table S3. LOO and WAIC values for model diagnostics. The smaller values of WAIC and LOO indicate better model descriptive performance.

**Model**	**LOO**	**WAIC**
1. Additive Error Model (AEM)	54978.8	54590.6
2. Combined Error Model (CEM)	52981.0	52574.0
3. CEM, V_m~ MWT, CL_{bo} ~ BWT	52893.0	52505.6
4. CEM, CEM ~ Country	49887.4	49731.8
5. CEM, CEM ~ Country, V_m~ MWT, CL_{bo} ~ BWT	50347.8	49931.0

CEM = combined error model (additive and exponential); V_m ~ MWT indicates inclusion of MWT (mother's weight) as a covariate on V_m and CL_{bo} ~ BWT indicates inclusion of BWT (baby's weight) as a covariate on CL_{bo}. CEM ~ Country indicates inclusion of country as a covariate on Error Model.

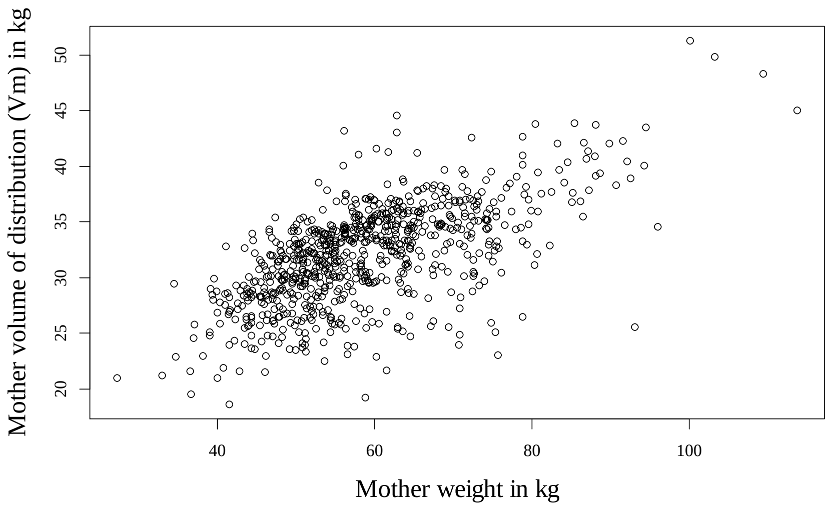

Figure S2. Correlation of mother volume of distribution V_m and mother's weight

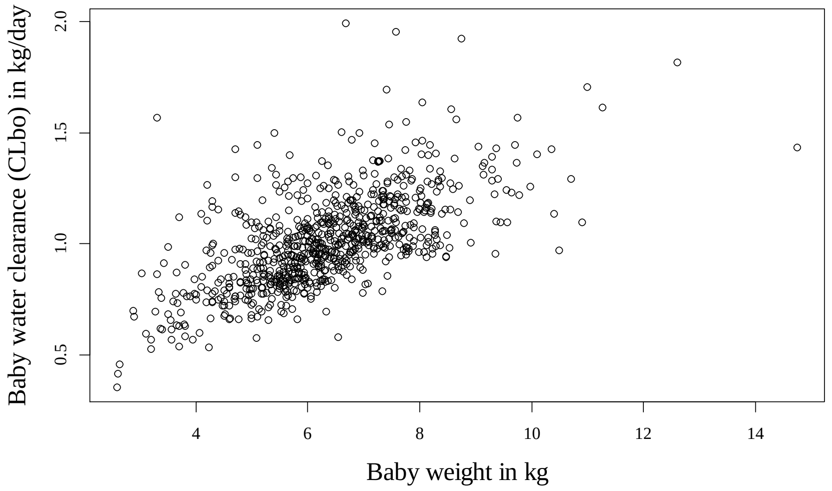

Figure S3. Correlation of baby water clearance CL_{bo} and baby's weight
