# Supplementary material for: Development of a Parsimonious Design for Optimal Classification of Exclusive Breastfeeding
Source: CPT Pharmacometrics Syst Pharmacol. 2019 Jul 3;8(8):596–605. doi: 10.1002/psp4.12428 (PMC6709417; doi:10.1002/psp4.12428)
Supplement: Supplementary file 6 — Supplementary Material S6. [file PSP4-8-596-s006.pdf]

Supplement 6.

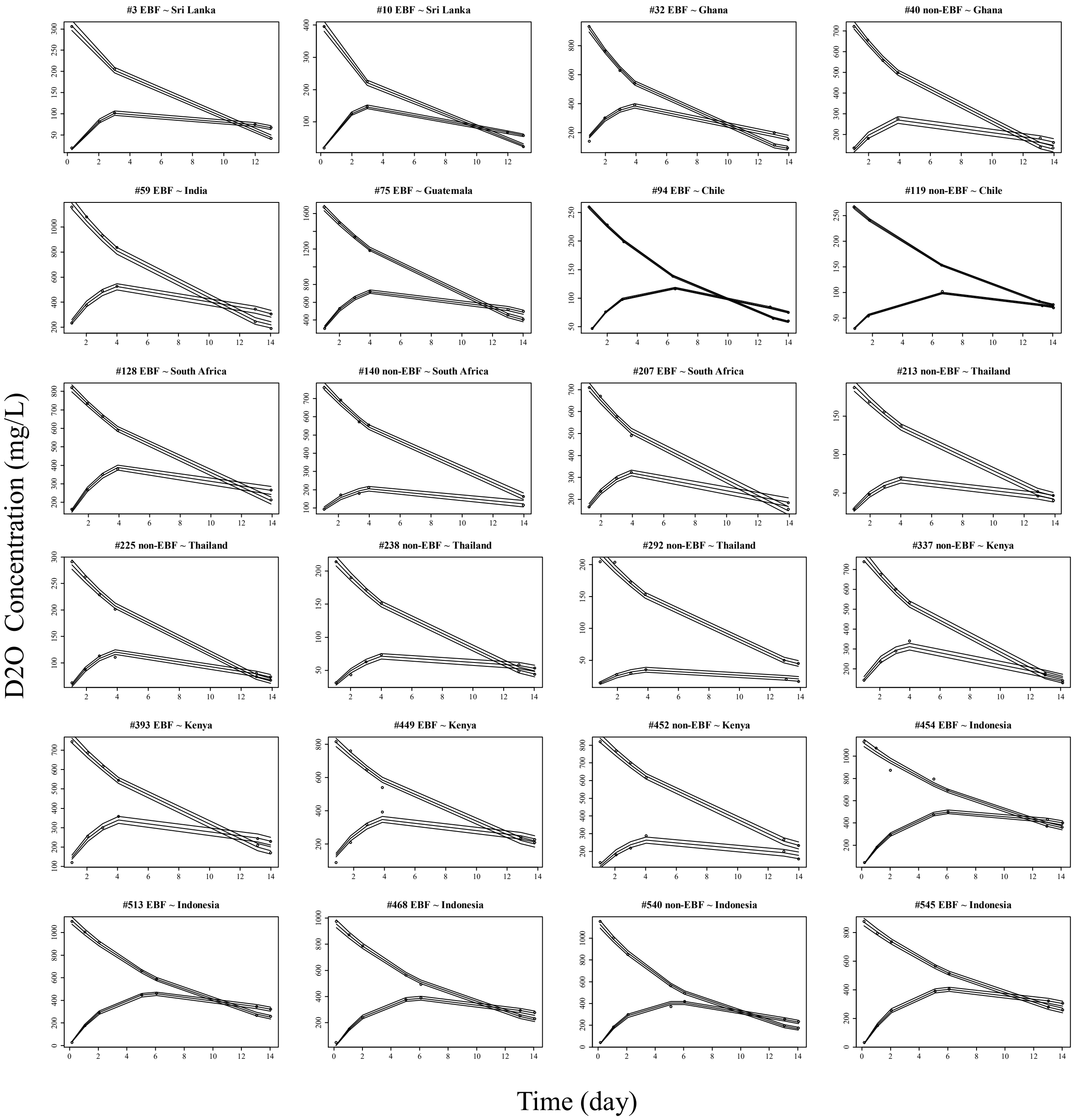

Figure S4 Individual Visual Predictive Check for the best final model evaluation. Open circles are the observations. The solid lines (median, 2.5% and 97.5% quantiles) are the model predicted response.

The model building data contains the calibration study data (i.e. well-controlled EBF mother-infant pairs) and the field studies (i.e. not-controlled pairs). Since all the samples around days 5 and 6 are from the calibration study data and the diagnostic value can be hampered if we plot the two data together. Therefore, the pcvc VPCs of the calibration study data (Figures S5 and S6) and the field study data (Figures S7 and S8) are plotted separately. It can be seen that the model describes the observations appropriately.

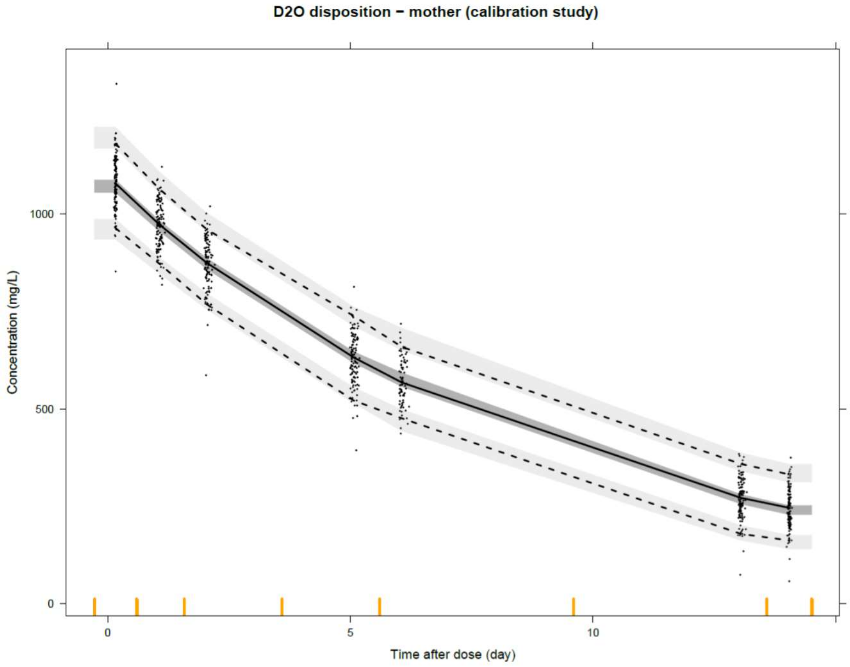

Figure S5. Prediction-corrected and variance-corrected VPCs of the best final model at population level for mothers in the calibration study data. The raw data are represented as *black spots* and *lines*. Grey shaded areas are the 90% confidence interval for the 5th, 50th and 95th percentile of the simulated data. Seven bins are used and the short yellow bars are the bin boundaries.

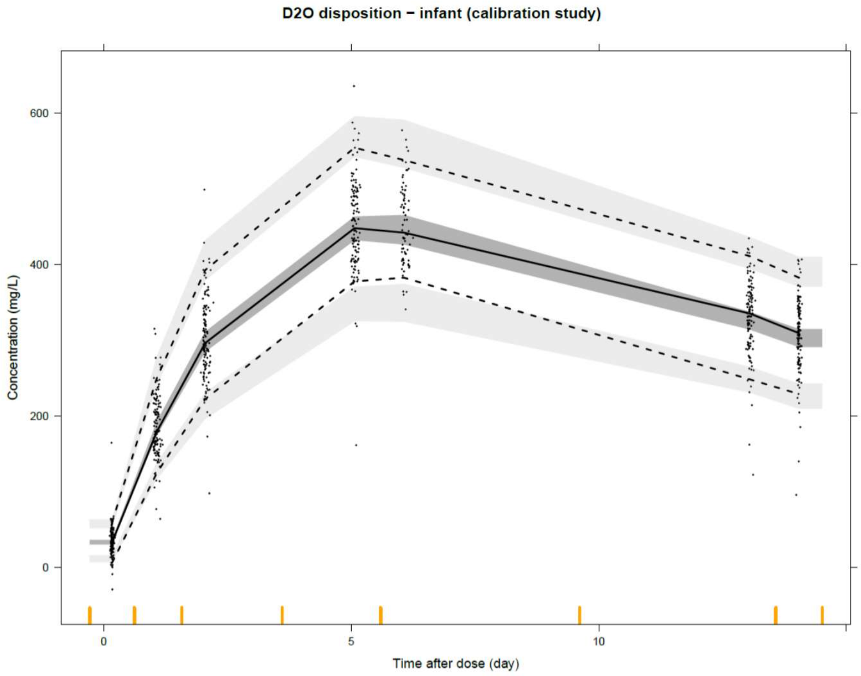

Figure S6. Prediction-corrected and variance-corrected VPCs of the best final model at population level for infants in the calibration study data. The raw data are represented as *black spots* and *lines*. Grey shaded areas are the 90% confidence interval for the 5th, 50th and 95th percentile of the simulated data. Seven bins are used and the short yellow bars are the bin boundaries.

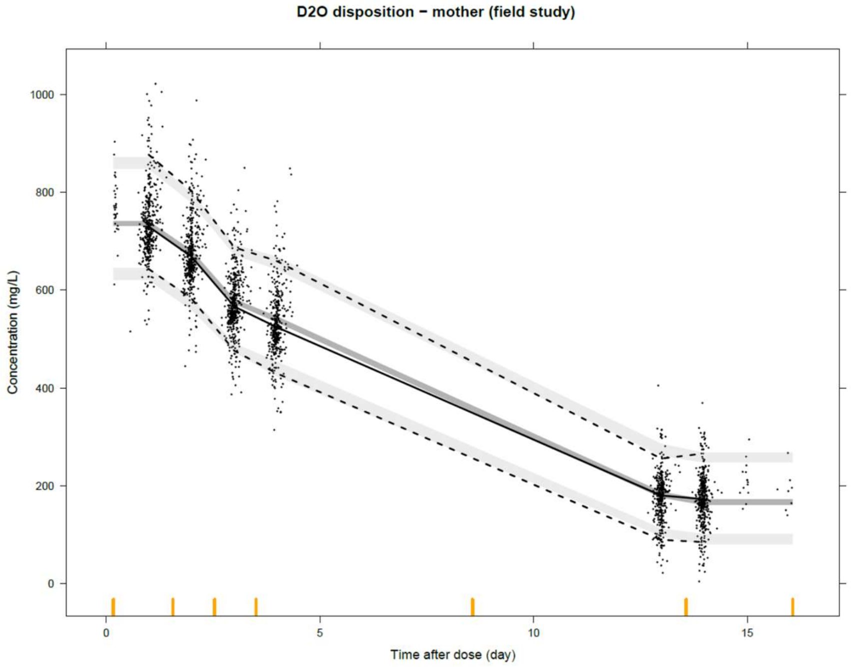

Figure S7. Prediction-corrected and variance-corrected VPCs of the best final model at population level for mothers in the field study data. The raw data are represented as *black spots* and *lines*. Grey shaded areas are the 90% confidence interval for the 5th, 50th and 95th percentile of the simulated data. Six bins are used and the short yellow bars are the bin boundaries.

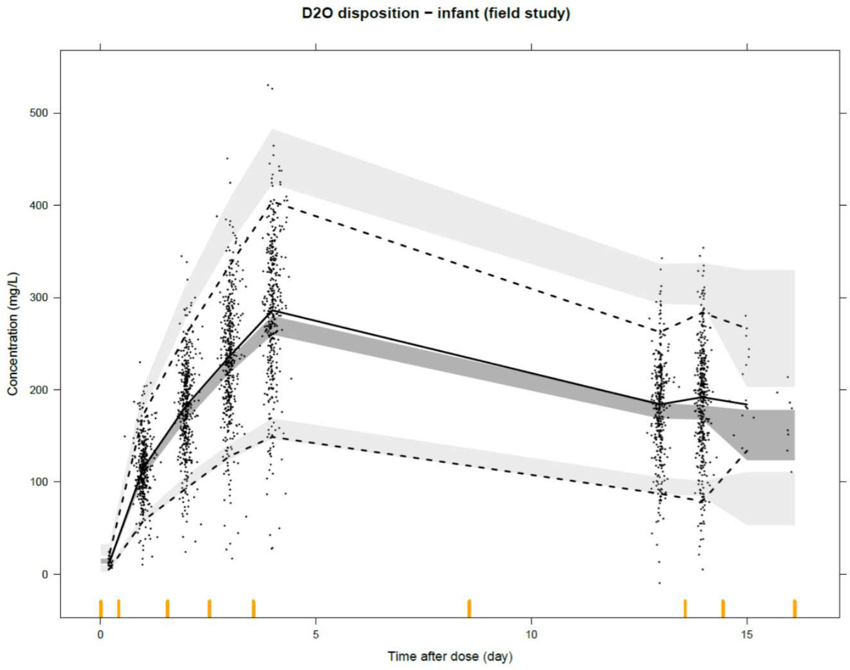

Figure S8. Prediction-corrected and variance-corrected VPCs of the best final model at population level for infants in the field study data. The raw data are represented as *black spots* and *lines*. Grey shaded areas are the 90% confidence interval for the 5th, 50th and 95th percentile of the simulated data. Seven bins are used and the short yellow bars are the bin boundaries.
