# Supplementary material for: Development of a Parsimonious Design for Optimal Classification of Exclusive Breastfeeding
Source: CPT Pharmacometrics Syst Pharmacol. 2019 Jul 3;8(8):596–605. doi: 10.1002/psp4.12428 (PMC6709417; doi:10.1002/psp4.12428)
Supplement: Supplementary file 7 — Supplementary Material S7. [file PSP4-8-596-s007.pdf]

Supplement 7.

The *model building data set* containing 2/3rd of the field data set and all of the calibration data set was used for selecting streamlined designs. It can be seen in Figure S9 that the sampling times of the *model building data set* were unevenly distributed and most mother-child pairs were sampled on days 0,1, 2, 3, 4, 5, 6, 13 and 14. For days 10, 11, 12, 15 and 16 there were too few actual measured samples on these days and hence the design would be based solely on imputation rather than a mixture of imputation and measured data. Therefore, days 10, 11, 12, 15 and 16 were not included to create *the imputed full data set*. Days 7, 8 and 9 were included in the *imputed full data set*, although with few actual measured samples occurred on these days. This is because the maximum information density of CL_{mb} and CL_{bo} occurs close to these days (see Figures S10 and S11), and hence these days are of particular importance in estimation of both parameters which are critical components for estimating R_s. Day 0 was not chosen to create the *imputed full data set*, as the model predicts that the passage of D_2O starts to occur immediately at the time of the dose to the mother. While this assumption has little influence after the first few hours it is not reasonable due to misspecification of D_2O kinetics in the first few hours post-dose.

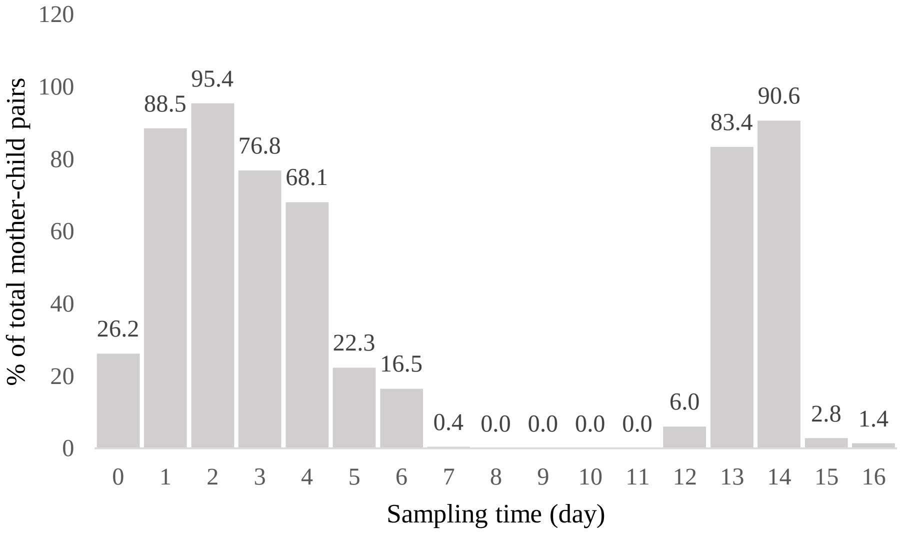

Figure S9. The percent of mother-child pairs who provided a sample on each day.

Resultantly, on the basis of days 1, 2, 3, 4, 5, 6, 7, 8, 9, 13 and 14, the *imputed full data set* was created. The missing observations were imputed with the posterior mean value of the data given the dose of D_2O, relevant covariates and sample time.

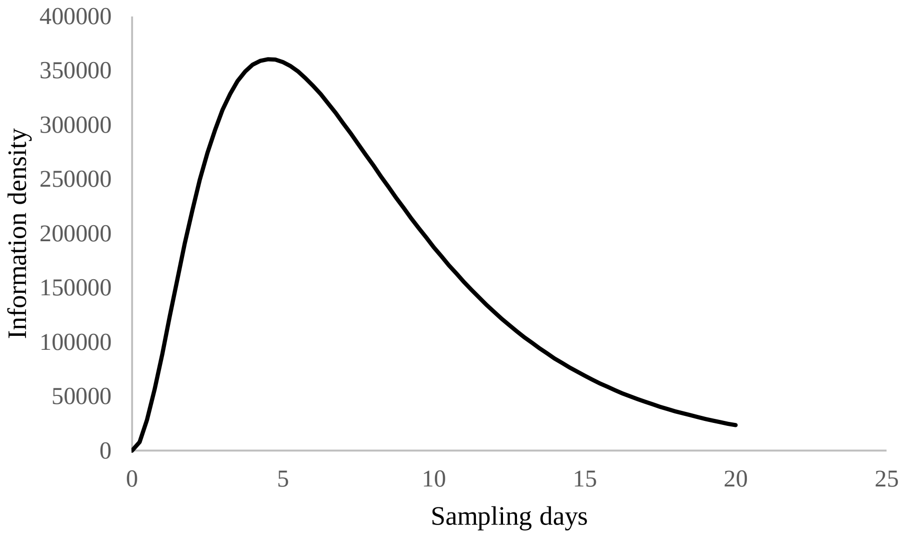

Figure S10. CL_{mb} information density against sampling time. The maximum information density occurs approximately on the day 4.5.

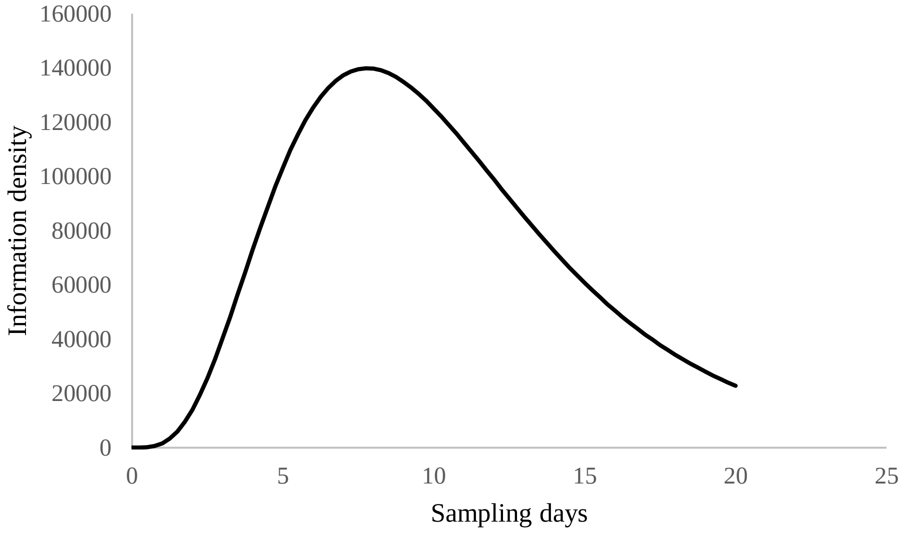

Figure S11. CL_{bo} information density against sampling time. The maximum information density occurs approximately on the day 8.
