# Supplementary material for: Development of a Parsimonious Design for Optimal Classification of Exclusive Breastfeeding
Source: CPT Pharmacometrics Syst Pharmacol. 2019 Jul 3;8(8):596–605. doi: 10.1002/psp4.12428 (PMC6709417; doi:10.1002/psp4.12428)
Supplement: Supplementary file 8 — Supplementary Material S8. [file PSP4-8-596-s008.pdf]

Supplement 8.

Table S4. *Streamline designs A* (the mixed prior) with successful convergence.

No	Design	Sensitivity	Specificity	No	Design	Sensitivity	Specificity
1	3+8	0.83	0.93	19	2+5+7	0.93	0.94
2	3+9	0.84	0.93	20	2+5+8	0.95	0.95
3	4+7	0.82	0.93	21	2+5+9	0.97	0.95
4	5+7	0.98	0.94	22	3+5+7	0.97	0.97
5	5+8	0.99	0.94	23	3+5+8	0.99	0.97
6	5+9	0.98	0.95	24	3+5+9	0.99	0.97
7	3+13	0.84	0.94	25	2+7+13	0.98	0.94
8	5+14	0.94	0.96	26	2+7+14	1.00	0.94
9	6+14	0.95	0.97	27	2+8+13	0.97	0.93
10	7+13	0.98	0.96	28	2+8+14	0.99	0.93
11	7+14	0.98	0.97	29	2+9+13	0.97	0.92
12	8+13	0.99	0.96	30	2+9+14	0.99	0.92
13	8+14	0.99	0.96	31	3+7+13	0.99	0.94
14	9+13	0.99	0.95	32	3+7+14	1.00	0.93
15	9+14	0.99	0.96	33	3+8+13	0.99	0.94
16	1+5+7	0.89	0.93	34	3+8+14	1.00	0.93
17	1+5+8	0.91	0.94	35	3+9+13	0.99	0.95
18	1+5+9	0.96	0.95	36	3+9+14	0.99	0.93

Table S5. *Streamline designs B* (3-sample and the mixed prior) with successful convergence.

No	Design	Sensitivity	Specificity	No	Design	Sensitivity	Specificity
1	1+2+4	0.19	1.00	14	2+3+4	0.02	0.99
2	1+2+5	0.67	0.99	15	2+3+5	0.67	0.98
3	1+2+6	0.81	0.98	16	2+3+6	0.82	0.98
4	1+2+7	0.88	0.97	17	2+3+7	0.89	0.97
5	1+3+4	0.00	1.00	18	2+4+5	0.57	0.98
6	1+3+5	0.50	0.99	19	2+4+6	0.76	0.97
7	1+3+6	0.76	0.98	20	2+4+7	0.84	0.97
8	1+3+7	0.86	0.98	21	2+5+6	0.87	0.92
9	1+4+5	0.48	0.98	22	2+6+7	0.93	0.92
10	1+4+6	0.70	0.97	23	3+4+5	0.55	0.99
11	1+4+7	0.81	0.97	24	3+4+6	0.78	0.98
12	1+5+6	0.82	0.88	25	3+5+6	0.94	0.96
13	1+6+7	0.87	0.88	26	3+6+7	0.97	0.95

Table S6. *Streamline designs B* (4-sample, 5-sample, 6-sample and 7-sample and the mixed prior) with successful convergence.

No	Design	Sensitivity	Specificity	No	Design	Sensitivity	Specificity
1	1+2+3+4	0.14	1.00	23	3+4+5+7	0.93	0.97
2	1+2+3+5	0.68	0.99	24	3+4+6+7	0.93	0.98
3	1+2+3+6	0.82	0.98	25	3+5+6+7	0.97	0.95
4	1+2+3+7	0.90	0.97	26	1+2+3+4+5	0.65	0.98
5	1+3+4+5	0.55	0.98	27	1+2+3+4+6	0.78	0.98
6	1+3+4+6	0.75	0.97	28	1+2+3+4+7	0.87	0.98
7	1+3+4+7	0.86	0.97	29	1+3+4+5+6	0.84	0.97
8	1+3+5+6	0.87	0.98	30	1+3+4+5+7	0.90	0.98
9	1+3+5+7	0.93	0.97	31	1+3+4+6+7	0.92	0.98
10	1+4+5+6	0.80	0.96	32	1+3+5+6+7	0.94	0.97
11	1+4+5+7	0.89	0.96	33	1+4+5+6+7	0.92	0.95
12	1+5+6+7	0.87	0.89	34	2+3+4+5+6	0.84	0.97
13	2+3+4+5	0.60	0.97	35	2+3+4+5+7	0.91	0.97
14	2+3+4+6	0.79	0.97	36	2+3+4+6+7	0.93	0.97
15	2+3+4+7	0.87	0.97	37	2+3+5+6+7	0.95	0.97
16	2+3+5+6	0.88	0.97	38	2+4+5+6+7	0.96	0.96
17	2+3+5+7	0.92	0.97	39	3+4+5+6+7	0.95	0.97
18	2+4+5+6	0.84	0.97	40	1+2+3+4+5+7	0.89	0.98
19	2+4+5+7	0.90	0.96	41	1+2+3+4+6+7	0.91	0.98
20	2+4+6+7	0.93	0.96	42	1+2+3+5+6+7	0.94	0.98
21	2+5+6+7	0.93	0.92	43	2+3+4+5+6+7	0.94	0.97
22	3+4+5+6	0.88	0.98	44	1+2+3+4+5+6+7	0.93	0.98

Table S7. *Streamline designs A* (the low information prior) with successful convergence.

No	Design	Sensitivity	Specificity	No	Design	Sensitivity	Specificity
1	3+7	0.80	0.89	18	2+5+7	0.96	0.96
2	3+9	0.82	0.90	19	2+5+8	0.96	0.96
3	5+8	0.87	1.00	20	2+5+9	0.95	0.94
4	5+9	0.90	0.99	21	3+5+7	0.97	0.97
5	3+13	0.68	1.00	22	3+5+8	0.97	0.97
6	3+14	0.78	0.95	23	3+5+9	0.96	0.98
7	5+13	0.91	0.94	24	2+7+13	0.99	0.95
8	6+14	0.92	0.96	25	2+7+14	0.98	0.96
9	7+13	0.99	0.95	26	2+8+13	0.99	0.92
10	7+14	0.99	0.96	27	2+8+14	0.94	0.93
11	8+13	0.92	0.98	28	2+9+13	0.94	0.95
12	8+14	0.96	0.99	29	2+9+14	0.99	0.96
13	9+13	0.98	0.92	30	3+7+13	0.96	0.96
14	9+14	0.97	0.93	31	3+7+14	0.93	0.95
15	1+5+7	0.89	0.96	32	3+8+13	0.96	0.91
16	1+5+8	0.92	0.93	33	3+8+14	0.93	0.99
17	1+5+9	0.97	0.94	34	3+9+13	0.99	0.90
				35	3+9+14	0.95	0.93

Table S8. *Streamline designs B* (3-sample and the low information prior) with successful convergence.

No	Design	Sensitivity	Specificity	No	Design	Sensitivity	Specificity
1	1+2+6	0.80	0.96	12	2+3+5	0.58	0.99
2	1+2+7	0.95	0.99	13	2+3+7	0.85	0.99
3	1+3+4	0.10	0.99	14	2+4+6	0.74	0.96
4	1+3+5	0.56	0.98	15	2+4+7	0.81	0.96
5	1+3+6	0.35	0.97	16	2+5+6	0.88	0.92
6	1+3+7	0.84	0.96	17	2+6+7	0.92	1.00
7	1+4+5	0.42	0.99	18	3+4+5	0.41	0.96
8	1+4+6	0.72	0.99	19	3+4+6	0.74	0.99
9	1+4+7	0.75	0.93	20	3+5+6	0.92	0.94
10	1+5+6	0.74	0.86	21	3+6+7	0.91	0.99
11	1+6+7	0.80	0.85				

Table S9. *Streamline designs B* (4-sample, 5-sample, 6-sample and 7-sample and the low information prior) with successful convergence.

No	Design	Sensitivity	Specificity	No	Design	Sensitivity	Specificity
1	1+2+3+4	0.14	1.00	22	3+4+6+7	0.89	0.96
2	1+2+3+5	0.68	0.99	23	3+5+6+7	0.87	0.99
3	1+2+3+6	0.82	0.98	24	1+2+3+4+5	0.65	0.99
4	1+2+3+7	0.90	0.97	25	1+2+3+4+6	0.63	0.99
5	1+3+4+5	0.55	0.98	26	1+2+3+4+7	0.75	1.00
6	1+3+4+6	0.75	0.97	27	1+3+4+5+6	0.74	1.00
7	1+3+4+7	0.86	0.97	28	1+3+4+5+7	0.68	0.99
8	1+3+5+7	0.95	0.99	29	1+3+4+6+7	0.90	0.94
9	1+4+5+6	0.81	0.99	30	1+3+5+6+7	0.91	0.99
10	1+4+5+7	0.85	0.98	31	1+4+5+6+7	0.90	0.98
11	1+5+6+7	0.88	0.92	32	2+3+4+5+6	0.82	0.96
12	2+3+4+6	0.85	0.93	33	2+3+4+5+7	0.82	0.97
13	2+3+4+7	0.85	0.92	34	2+3+4+6+7	0.91	0.98
14	2+3+5+6	0.84	0.93	35	2+3+5+6+7	0.95	0.95
15	2+3+5+7	0.90	0.99	36	2+4+5+6+7	0.97	0.97
16	2+4+5+6	0.78	0.99	37	3+4+5+6+7	0.96	0.96
17	2+4+5+7	0.86	0.95	38	1+2+3+4+5+7	0.87	0.96
18	2+4+6+7	0.92	0.98	39	1+2+3+4+6+7	0.88	0.99
19	2+5+6+7	0.93	0.96	40	1+2+3+5+6+7	0.92	0.99
20	3+4+5+6	0.80	0.96	41	2+3+4+5+6+7	0.92	0.99
21	3+4+5+7	0.93	0.97	42	1+2+3+4+5+6+7	0.92	0.98

Table S10. *Streamline designs A* (the informative prior) with successful convergence.

No	Design	Sensitivity	Specificity	No	Design	Sensitivity	Specificity
1	3+8	0.83	0.93	19	2+5+7	0.93	0.94
2	3+9	0.84	0.93	20	2+5+8	0.95	0.95
3	4+7	0.82	0.93	21	2+5+9	0.97	0.95
4	5+7	0.98	0.94	22	3+5+7	0.97	0.97
5	5+8	0.99	0.94	23	3+5+8	0.99	0.97
6	5+9	0.98	0.95	24	3+5+9	0.99	0.97
7	3+13	0.84	0.94	25	2+7+13	0.98	0.94
8	5+14	0.94	0.96	26	2+7+14	1.00	0.94
9	6+14	0.95	0.97	27	2+8+13	0.97	0.93
10	7+13	0.98	0.96	28	2+8+14	0.99	0.93
11	7+14	0.98	0.97	29	2+9+13	0.97	0.92
12	8+13	0.99	0.96	30	2+9+14	0.99	0.92
13	8+14	0.99	0.96	31	3+7+13	0.99	0.94
14	9+13	0.99	0.95	32	3+7+14	1.00	0.93
15	9+14	0.99	0.96	33	3+8+13	0.99	0.94
16	1+5+7	0.89	0.93	34	3+8+14	1.00	0.93
17	1+5+8	0.91	0.94	35	3+9+13	0.99	0.95
18	1+5+9	0.96	0.95	36	3+9+14	0.99	0.93

Table S11. *Streamline designs B* (3-sample and the informative prior) with successful convergence.

No	Design	Sensitivity	Specificity	No	Design	Sensitivity	Specificity
1	1+2+4	0.19	1.00	14	2+3+4	0.02	0.99
2	1+2+5	0.67	0.99	15	2+3+5	0.67	0.98
3	1+2+6	0.81	0.98	16	2+3+6	0.82	0.98
4	1+2+7	0.88	0.97	17	2+3+7	0.89	0.97
5	1+3+4	0.00	1.00	18	2+4+5	0.57	0.98
6	1+3+5	0.50	0.99	19	2+4+6	0.76	0.97
7	1+3+6	0.76	0.98	20	2+4+7	0.84	0.97
8	1+3+7	0.86	0.98	21	2+5+6	0.87	0.92
9	1+4+5	0.48	0.98	22	2+6+7	0.93	0.92
10	1+4+6	0.70	0.97	23	3+4+5	0.55	0.99
11	1+4+7	0.81	0.97	24	3+4+6	0.78	0.98
12	1+5+6	0.82	0.88	25	3+5+6	0.94	0.96
13	1+6+7	0.87	0.88	26	3+6+7	0.97	0.95

Table S12. *Streamline designs B* (4-sample, 5-sample, 6-sample and 7-sample and the informative prior) with successful convergence.

No	Design	Sensitivity	Specificity	No	Design	Sensitivity	Specificity
1	1+2+3+4	0.14	1.00	23	3+4+5+7	0.93	0.97
2	1+2+3+5	0.68	0.99	24	3+4+6+7	0.93	0.98
3	1+2+3+6	0.82	0.98	25	3+5+6+7	0.97	0.95
4	1+2+3+7	0.90	0.97	26	1+2+3+4+5	0.65	0.98
5	1+3+4+5	0.55	0.98	27	1+2+3+4+6	0.78	0.98
6	1+3+4+6	0.75	0.97	28	1+2+3+4+7	0.87	0.98
7	1+3+4+7	0.86	0.97	29	1+3+4+5+6	0.84	0.97
8	1+3+5+6	0.87	0.98	30	1+3+4+5+7	0.90	0.98
9	1+3+5+7	0.93	0.97	31	1+3+4+6+7	0.92	0.98
10	1+4+5+6	0.80	0.96	32	1+3+5+6+7	0.94	0.97
11	1+4+5+7	0.89	0.96	33	1+4+5+6+7	0.92	0.95
12	1+5+6+7	0.87	0.89	34	2+3+4+5+6	0.84	0.97
13	2+3+4+5	0.60	0.97	35	2+3+4+5+7	0.91	0.97
14	2+3+4+6	0.79	0.97	36	2+3+4+6+7	0.93	0.97
15	2+3+4+7	0.87	0.97	37	2+3+5+6+7	0.95	0.97
16	2+3+5+6	0.88	0.97	38	2+4+5+6+7	0.96	0.96
17	2+3+5+7	0.92	0.97	39	3+4+5+6+7	0.95	0.97
18	2+4+5+6	0.84	0.97	40	1+2+3+4+5+7	0.89	0.98
19	2+4+5+7	0.90	0.96	41	1+2+3+4+6+7	0.91	0.98
20	2+4+6+7	0.93	0.96	42	1+2+3+5+6+7	0.94	0.98
21	2+5+6+7	0.93	0.92	43	2+3+4+5+6+7	0.94	0.97
22	3+4+5+6	0.88	0.98	44	1+2+3+4+5+6+7	0.93	0.98
